# Analysis of polymorphisms in 16 genes in type 1 diabetes that have been associated with other immune-mediated diseases

**DOI:** 10.1186/1471-2350-7-20

**Published:** 2006-03-06

**Authors:** Deborah J Smyth, Joanna MM Howson, Felicity Payne, Lisa M Maier, Rebecca Bailey, Kieran Holland, Christopher E Lowe, Jason D Cooper, John S Hulme, Adrian Vella, Ingrid Dahlman, Alex C Lam, Sarah Nutland, Neil M Walker, Rebecca CJ Twells, John A Todd

**Affiliations:** 1 Juvenile Diabetes Research Foundation/Wellcome Trust Diabetes and Inflammation Laboratory, Cambridge Institute for Medical Research, Wellcome Trust/MRC Building, Addenbrooke's Hospital, Hills Rd, Cambridge, CB2 2XY, UK

## Abstract

**Background:**

The identification of the HLA class II, insulin (INS), CTLA-4 and PTPN22 genes as determinants of type 1 diabetes (T1D) susceptibility indicates that fine tuning of the immune system is centrally involved in disease development. Some genes have been shown to affect several immune-mediated diseases. Therefore, we tested the hypothesis that alleles of susceptibility genes previously associated with other immune-mediated diseases might perturb immune homeostasis, and hence also associate with predisposition to T1D.

**Methods:**

We resequenced and genotyped tag single nucleotide polymorphisms (SNPs) from two genes, *CRP *and *FCER1B*, and genotyped 27 disease-associated polymorphisms from thirteen gene regions, namely *FCRL3*, *CFH*, *SLC9A3R1*, *PADI4*, *RUNX1, SPINK5*, *IL1RN*, *IL1RA*, *CARD15*, *IBD5*-locus (including *SLC22A4*), *LAG3*, *ADAM33 *and *NFKB1*. These genes have been associated previously with susceptibility to a range of immune-mediated diseases including rheumatoid arthritis (RA), systemic lupus erythematosus (SLE), Graves' disease (GD), psoriasis, psoriatic arthritis (PA), atopy, asthma, Crohn disease and multiple sclerosis (MS). Our T1D collections are divided into three sample subsets, consisting of set 1 families (up to 754 families), set 2 families (up to 743 families), and a case-control collection (ranging from 1,500 to 4,400 cases and 1,500 to 4,600 controls). Each SNP was genotyped in one or more of these subsets. Our study typically had approximately 80% statistical power for a minor allele frequency (MAF) >5% and odds ratios (OR) of 1.5 with the type 1 error rate, α = 0.05.

**Results:**

We found no evidence of association with T1D at most of the loci studied 0.02 <*P *< 1.0. Only a SNP in *ADAM33*, rs2787094, was any evidence of association obtained, *P *= 0.0004 in set 1 families (relative risk (RR) = 0.78), but further support was not observed in the 4,326 cases and 4,610 controls, *P *= 0.57 (OR = 1.02).

**Conclusion:**

Polymorphisms in a variety of genes previously associated with immune-mediated disease susceptibility and/or having effects on gene function and the immune system, are unlikely to be affecting T1D susceptibility in a major way, even though some of the genes tested encode proteins of immune pathways that are believed to be central to the development of T1D. We cannot, however, rule out effect sizes smaller than OR 1.5.

## Background

The four susceptibility loci identified so far in T1D, the HLA class II gene complex [1], *INS *[2], *CTLA4 *[3] and *PTPN22 *[4,5] indicate that the regulation of T cell development, activation, expansion and homeostasis is a central component of disease development. A fifth locus, the *IL2R2/CD25 *region [6] awaits independent replication and fine mapping of the aetiological variant. With the exception of *INS *[7], these genes contain polymorphisms that are associated with susceptibility to other immune-mediated diseases.

Therefore, we hypothesised that further susceptibility variants for T1D may reside in genes previously associated with other immune-mediated diseases, as prior evidence suggests the presence of shared disease susceptibility genes. For example, in families with T1D, other immune-mediated diseases, such as RA and autoimmune thyroid disease (AITD), occur more frequently than expected by chance, indicative of a partly shared genetic basis [7]. This model has gained significant support recently with the association of the Arg620Trp non-synonymous SNP in the PTPN22 gene, encoding the lymphoid specific phosphatase, LYP; not only with T1D [4] but also with GD, RA and SLE [5,8,9]. Likewise *CTLA4 *has been associated with T1D, AITD, RA and SLE [3,10].

Furthermore, as susceptibility to T1D and other autoimmune diseases is probably directly related to the homeostatic, regulatory state of the immune system, it is possible that variants in immune response genes influence susceptibility to T1D via alteration of networks of immune regulation. For example, the CARD15 gene product, NOD2, influences the development of the adaptive immune response [11,12] and functional variants of the gene predispose to the inflammatory bowel disease (IBD), Crohn disease (Table 1). Both the Th1 and mucosal immune system are thought to be important in T1D aetiology [13].

**Table 1:** Previously associated polymorphisms with other immune-mediated diseases and references.

**Gene (locus link id)**	**Gene Function**	**Polymorphisms**	**MAF**	**Reference and previous association**
*CRP *1q21-q23 (1401)	Activates the classical pathway of complement. SNP2 alters basal levels of CRP. SNP4 has been associated with SLE and antinuclear autoantibody production. A polymorphic GT repeat in *CRP *has been associated with SLE.	SNP2 (rs1800947) SNP4 (rs1205) Microsatellite (ss28514831)	0.07 & 0.33 respectively, Caucasian parental. GT^16 ^& GT^21^, 0.62 and 0.24, respectively, Caucasian controls.	SLE: Microsatellite *P *= 0.007, SNP4 *P *= 0.0008; 586 families [14]. Microsatellite [15].

*FCRL3 *1q21-q22 (115352)	*FCRL3*, a member of the Fc receptor-like family, polymorphism alters the binding affinity of nuclear factor κB and regulates *FCRL3 *expression. Associated with RA, SLE and autoimmune thyroid disease (GD and HT)	Fcrl3_3 (rs7528684)	0.37 Japanese controls	RA: *P *= 8.5 × 10^-7^, OR = 2.15 (95% CI = 1.58–2.93) 830 cases and 658 controls. SLE: *P *= 0.0017, OR = 1.49 (95% CI = 1.16–1.92) 564 cases. GD: *P *= 7.4 × 10^-5^, OR = 1.79 (95% CI = 1.34–2.39) 351 cases. HT: *P *= 0.022, OR = 1.62 (95% CI = 1.07–2.47) 158 cases [43].

*CFH *1q32 (3075)	Complement factor H, a key regulator of the complement system of innate immunity, binds heparin and CRP	His402Tyr (rs1061170)	0.41 controls, white, not of Hispanic origin	Age-related macular degeneration: (nominal *P *= <10^-7^) 96 cases and 50 controls [44].

*PAD14 *1q36.13 (23569)	Peptidylarginine deiminases role in granulocyte & macrophage development, leading to inflammation and immune response, associated with RA.	PADI4-94 (rs2240340)	0.37 Japanese controls	RA: *P *= 8 × 10^-6^, OR = 1.97 (95% CI = 1.44–2.69) 830 cases and 736 controls [45].

*IL1RN & IL1A *2q14 (3557 & 3552)	Cytokines involved in the inflammatory response, polymorphisms confer susceptibility to RA and Ankylosing spondylitis.	IL1RN+2017 (rs2419598) IL1A-889 (rs1800587)	0.22 & 0.27, respectively, Caucasian controls	RA: *P *= 0.008; 406 Dutch cases and 245 controls [46]. Ankylosing spondylitis: *P *= 0.025; 227 British families, 317 parent-case trios and 200 controls [47].

*NFKB1 *4q24 (4790)	*NFκB *is a transcription regulator that is activated by various intra- and extra-cellular stimuli such as cytokines, oxidant-free radicals, ultraviolet irradiation, and bacterial or viral products. Inappropriate activation of *NFκB *has been associated with a number of inflammatory diseases	(CA) dinucleotide repeat microsatellite	Allele 8, A10 & A14, 0.19, 0.02 and 0.28 respectively, UK controls	T1D: A10: *P *= 0.000001, OR = 9.4; 434 cases, 222 controls [36]. T1D: no association; 236 Danish families [37].

*SLC22A4 *5q31 (6583)	The encoded protein is an organic cation transporter and plasma integral membrane protein containing eleven putative transmembrane domains as well as a nucleotide-binding site motif. Polymorphisms confer susceptibility to RA.	SLC22A4:F1 (rs2073838) SLC22A4:F2 (rs3792876)	0.31 & 0.32 respectively, Japanese controls	RA: *P *= 0.000034, OR = 1.98 (95% CI = 1.43–2.75) 830 cases and 658 controls [48]. CD: *P *= 0.001, OR = 2.1 (95% CI = 1.31–3.39) 203 cases and 200 controls [49].

*IBD5 *locus 5q31 (50941)	Confers susceptibility to Crohn disease.	IGR2198 (rs11739135)	0.36 Canadian parental.	CD: *P *= 0.000048; 256 trios [50].

*SPINK5 *5q32 (11005)	Encodes a 15 domain serine proteinase inhibitor (*LEKTI*) involved in anti-inflammatory and/or antimicrobial protection of mucous epithelial, polymorphisms associated with atopy, Netherton disease.	316G>A (ss28514851) 1103A>G (rs2303064) 1156G>A (rs2303063) 1258G>A (rs2303067) 2475G>T (rs2303070) 2915A>G (ss28514856)	0.03, 0.50, 0.13, 0.48, 0.08 & 0.04, respectively, UK controls	Atopy, Netherton disease: (rs2303064): *P *= 0.008, (rs2303067): *P *= 0.002; 148 families [51]. Asthma (rs2303067): *P *= 0.04, OR = 1.77 (95% CI = 1.02–3.06) 1161 children. Asthma and atopy: *P *= 0.007, OR = 4.56 (95% CI = 1.37–15.12) 37 German cases and 415 controls [52].

*FCER1B *11q13 (2206)	Encodes the beta subunit of the high affinity IgE receptor, a member of the membrane-spanning 4A gene family, and displays unique expression patterns among hematopoietic cells and nonlymphoid tissues. Responsible for initiating the allergic response, associated with atopy and atopic asthma.	Gly237Glu (rs569108)	0.06 Japanese controls	Atopy: two-point lod score 9.35 [17]. Childhood asthma (rs569108): *P *= <0.002, OR = 3; 200 Japanese cases and 100 controls [16].

*LAG3 *12p13.32 (3902)	Lymphocyte-activation protein 3 belongs to Ig superfamily and contains 4 extracellular Ig-like domains.	Thr455Ile (rs870849)	N/A	MS: *P *= 0.005; 576 cases and 662 controls [53].

*CARD15 *16q12 (64127)	Intracellular sensors of bacterial peptidoglycan These SNPs encode amino acid changes located in or near the leucine-rich repeat region, which is involved in peptidoglycan binding, conferring an increased risk of Crohn disease, PA and Blau syndrome.	SNP8 (rs2066844) SNP12 (rs2066845) SNP13 (ss28514842)	0.04, 0.01 & 0.02, respectively, Caucasian controls	CD (SNP13): *P *= 6 × 10^-6^; 235 families [54]. *P *= 0.0046; 416 families[55]. PA: *P *= 0.0027, OR = 3.5 (95% CI = 1.51–7.01) [56]. Blau Syndrome [57].

*SLC9A3R1 *17q25 (9368)	Immune synapse formation in T cells, polymorphism associated with psoriasis.	*SLC9A3R1 *(rs734232)	0.42 European controls	Psoriasis: *P *= 0.0009; 134 trios [58].

*ADAM33 *20p13 (80332)	Encodes a disintegrin and metalloprotease (ADAM) domain 33, which is a member of the ADAM protein family. Has a role in cell-cell and cell-matrix interactions, including fertilization, muscle development, and neurogenesis. Reported as an asthma and bronchial hyper-responsiveness susceptibility locus.	ST+4 (rs44707) V4 (rs2787094) Q-1 (rs612709) ST+7 (rs574174) T+1 (rs2280089) T2 (rs2280090)	0.48, 0.25, 0.24, 0.22, 0.08, 0.07 UK & USA controls	Asthma and bronchial hyperresponsiveness: *P *= 0.03 – 0.02; 130 cases and 217 controls [35]. *P *= 0.04–0.0009 in ethnically diverse populations [59].

*RUNX1 *21q22.3 (861)	The *RUNX1 *transcription factor is expressed mainly in hematopoietic cells and functions both to activate and to repress transcription through interactions with cofactors. This SNP alters a binding site for *RUNX1 *and has been associated with RA.	*RUNX1 *(rs2268277)	0.37 Japanese controls	RA: *P *= 0.0013, OR= 1.28 (95% CI = 1.10–1.48) 719 cases and 441 controls [48].

MAF: minor allele frequency, SLE: systemic lupus erythematosus, RA: rheumatoid arthritis, GD: Graves' disease, HT: Hashimoto thyroiditis, T1D: type 1 diabetes, CD: Crohn disease, MS: multiple sclerosis, PA: Psoriatic arthritis, OR: odds ratio, 95% CI: 95% confidence intervals, N/A: not available

The aim of this study was to determine whether previously associated polymorphisms of immune-mediated disease also predispose to T1D. We genotyped a total of 41 polymorphisms, including three microsatellite markers, from 16 genes, shown in Table 1, in large T1D collections. For two genes *C-reactive protein *(*CRP*), a marker for inflammation and associated with susceptibility to SLE [14,15], and *FCER1B*, high-affinity receptor for immunoglobulin E (IgE) (*MS4A2*), located in the putative T1D locus *IDDM4*, and associated with atopic illness [16,17], we also carried out a re-sequencing effort, to gain a more comprehensive profile of allelic variation of the genes and their potential association with T1D. Our sample sizes had good statistical power for ORs greater than 1.5 (Additional File 1) [18].

## Methods

### Subjects

T1D families were white European or of white European descent, with two parents and at least one affected child comprising DNA samples from up to 476 multiplex Diabetes UK Warren 1families [19], 278 multiplex HBDI families [20], 250 simplex Northern Ireland families [21], 260 simplex Norwegian families and 233 simplex Romanian families with inclusion criteria as reported in Vella *et al*. [22]. The T1D cases [23]and the 1958 BBC controls [24] have been described previously [5]. All DNA samples were collected after approval from the relevant research ethics committees and written informed consent was obtained from the participants. This project has run over a number of years during which samples of cases were still being collected. Consequently, owing to availability of DNA, polymorphisms were genotyped either in "set 1" families (n = 754 UK and USA multiplex families), "set 2" families (n = 743 Norwegian, Romanian and Northern Irish simplex families) and/or a British case-control collection consisting of between approximately 1,500 and 4,400 cases and 1,500 and 4,600 controls.

### SNP identification and genotyping

*CRP *(EMBL Accession number AL445528) and *FCER1B *(AP001181) were annotated locally, importing Ensembl information into a temporary ACeDB database as described previously [25]. After confirmation of gene structures by BLAST analysis, these were re-extracted in GFF format and submitted to T1Dbase [26].

Direct sequencing of nested PCR products from 32 T1D individuals was carried out for all exons of *CRP *and 3 kb 5' and 3' of the gene, using Applied Biosystems 3700 capillary sequencer. Polymorphisms were identified using the Staden Package and loaded into T1Dbase. *FCER1B *was also sequenced, as for *CRP*, in 96 T1D individuals for 2 kb 5' and 3 kb 3' of the gene and all exons, except exon 6, were successfully sequenced.

SNPs were genotyped using either TaqMan MGB chemistry (Applied Biosystems), Invader Biplex assay (Third Wave Technologies, Madison) [3] or PCR RFLP. The microsatellites were genotyped on an ABI3700 using fluorescent primers. All genotyping data were double scored to minimize error.

Family studies of variants with a MAF less than 5% may be compromised by apparent under transmission of alleles resulting from undetected genotyping errors [27]. To evaluate potential genotyping errors we genotyped a large selection of samples from three SNPs: SNP8 (rs2066844), SNP12 (rs2066845), SNP13 (ss28514842), twice, using two different methods, either TaqMan, Invader or PCR RFLP. The concordance rate between the methods was >99.2%.

### Statistical analyses

All statistical analyses were performed within *STATA *[28] making specific use of the *Genassoc *and *htSNP2 *packages for association and tag SNP selection, available from [29]. All genotyping data of unaffected parents and controls were assessed for, and found to be in Hardy-Weinberg equilibrium (*P *> 0.05). Associations of the SNPs and microsatellites were tested by the Transmission/disequilibrium test in the families. Allelic and genotypic relative risks were calculated using pseudo-controls and cases with conditional logistic regression [30]. In order to minimise any confounding due to variation in allele frequencies across Great Britain [31], case-control data were stratified by broad geographical region within the logistic regression model used to calculate ORs and test associations. Both genotypic and allelic effects were allowed for by modelling loci as three-level, 2 degrees of freedom (2 df), categorical variables corresponding to a model assuming no particular mode of inheritance and continuous (1 df) variables equating to a multiplicative model. Where a difference was found, by a likelihood ratio test, between the multiplicative model and a model assuming no particular mode of inheritance, the 2 df *P*-value is reported. The tag SNPs were analysed by use of a multivariate test statistic [6,32].

## Results

For *CRP *and *FCER1B*, we resequenced and followed a tag selection approach, selecting tags that capture the variation of the remaining common SNPs (MAF ≥ 0.05) with a minimum *R*^2 ^of 0.8 [32]. Nineteen SNPs were identified in *CRP*, five of which were novel (Additional File 2) [26] and seven tag SNPs were selected and genotyped in set 1 families (multilocus *P *= 0.49). We also genotyped the tag SNPs in the case-control collection (1,607 cases and 1,636 controls) and found no support for association (multilocus *P *= 0.42), indicating that common variants of *CRP *are unlikely to influence T1D susceptibility in a major way. The *CRP *intronic microsatellite (GT)_n _polymorphism (ss28514831) (Table 1 and Additional file 2) was also genotyped in set 1 and 2 families but showed no association with T1D (*P *= 0.90).

On resequencing *FCER1B*, which is located in the putative diabetes-susceptibility region *IDDM4 *[33], we identified 34 SNPs, 17 of which were novel, and selected five tag SNPs (Additional File 3) [26]. We were unable to detect the non-synonymous SNP in exon 7, Gly237Glu, in 96 DNA samples, that has previously been associated with atopic asthma [16]. We initially genotyped the five tag SNPs in set 1 families (multilocus *P *= 0.085), and then followed this result up in set 2 families giving a combined multilocus *P *= 0.070 (adjusted for two-stage design). In 1,600 cases and 1,636 controls we obtained *P *= 0.24, and the combination of the multilocus family and case-control results indicated that variants of *FCER1B *are unlikely to play a major role in T1D susceptibility (*P *= 0.23). The *FCER1B *intronic microsatellite polymorphism (ss28514807) also showed no evidence of association with T1D in set 1 and 2 families (*P *= 0.65).

Five SNPs from *FCRL3, CFH, SLC9A3R1, PADI4, RUNX1 *and six SNPs from *SPINK5 *were genotyped in set 1 families and a minimum of 1,500 cases and 1,500 controls (Table 2). All except *FCRL3 *(TDT *P *= 0.04) and one locus in *SPINK5 *(*P *= 0.03), showed a *P *value of > 0.05. However, we did not obtain any further evidence of association of *FCRL3 *in 1,896 cases and 2,020 controls (Table 2). *IL1RN, IL1RA, IGR2198 *and the *CARD15 *SNPs 8, 12 and 13 were genotyped in sets 1 and 2 families, and all showed *P *> 0.10 (Table 3). The two *SLC22A4 *SNPs and the single *LAG3 *SNP were genotyped in a minimum of 3,290 cases and 3,549 controls and showed no evidence of association with T1D (Table 3).

**Table 2:** Association analyses in type 1 diabetes families and case-control sample sets for immune-mediated disease associated polymorphisms.

Published SNP	Set 1 families					Cases and controls				
	MAF	N parent-child trios (T/NT)	P_TDT_	Allelic RR [95% CI]	Genotype RR [95% CI]	MAF	N cases/controls	P_1df_	Allelic OR [95% CI]	Genotype OR [95% CI]

*FCRL3 *A>G rs7528684	0.46	901 (641/571)	0.04	0.89 [0.80–1.00]	A/A 1.00 [ref] A/G 0.88 [0.75–1.04] G/G 0.79 [0.63–1.00]	0.45	1896/2020	0.97	1.00 [0.91–1.10]	A/A 1.00 [ref] A/G 1.02 [0.87–1.18] G/G 0.99 [0.82–1.20]
*CFH *A>G rs1061170	0.38	823 (541/517)	0.46	0.96 [0.85–1.08]	A/A 1.00 [ref] A/G 1.00 [0.85–1.17] G/G 0.88 [0.68–1.14]	0.38	3149/3485	0.87	0.99 [0.92–1.07]	A/A 1.00 [ref] A/G 1.07 [0.96–1.19] G/G 0.94 [0.81–1.10]
*SLC9A3R1 *G>A rs734232	0.45	965 (644/642)	0.96	1.00 [0.90–1.12]	G/G 1.00 [ref] G/A 1.03 [0.88–1.21] A/A 1.00 [0.80–1.25]	0.45	1578/1736	0.34	1.05 [0.95–1.17]	G/G 1.00 [ref] G/A 0.92 [0.78–1.10] A/A 1.14 [0.92–1.41]
*PADI4 *C>T rs2240340	0.41	942 (610/655)	0.21	1.07 [0.96–1.20]	T/T 1.00 [ref] C/T 1.07 [0.92–1.25] C/C 1.15 [0.92–1.44]	0.42	1573/1732	0.87	1.01 [0.91–1.12]	T/T 1.00 [ref] C/T 1.09 [0.92–1.29] C/C 0.99 [0.80–1.23]
*RUNX1 *C>G rs2268277	0.34	896 (578/565)	0.70	1.02 [0.91–1.15]	T/T 1.00 [ref] T/C 0.96 [0.83–1.11] C/C 1.12 [0.87–1.43]	0.36	1586/1725	0.21	0.93 [0.84–1.04]	T/T 1.00 [ref] T/C 0.90 [0.76–1.05] C/C 0.90 [0.71–1.14]
*SPINK5*										
+316 A>C ss28514851	0.04	178 (89/91)	0.88	0.98 [0.73–1.31]	A/A 1.00 [ref] A/C 0.98 [0.73–1.31] C/C 0.98 [0.10–9.55]	0.03	1540/1678	0.40	0.88 [0.66–1.18]	A/A 1.00 [ref] A/C 0.88 [0.65–1.19] C/C 0.82 [0.14–4.71]
+1103 T>C rs2303064	0.48	915 (583/602)	0.58	0.97 [0.86–1.09]	T/T 1.00 [ref] T/C 0.99 [0.84–1.17] C/C 0.94 [0.74–1.18]	0.47	1527/1665	0.30	0.95 [0.85–1.05]	T/T 1.00 [ref] T/C 0.96 [0.80–1.14] C/C 0.89 [0.72–1.10]
+1156 G>A rs2303063	0.10	357 (196/213)	0.40	0.92 [0.76–1.12]	G/G 1.00 [ref] G/A 0.93 [0.75–1.15] A/A 0.81 [0.42–1.57]	0.11	1530/1507	0.03 2 df	0.87 [0.73–1.04]	G/G 1.00 [ref] G/A 0.79 [0.64–0.96] A/A 1.48 [0.72–3.03]
+1258 C>T rs2303067	0.48	947 (617/622)	0.89	0.99 [0.89–1.11]	C/C 1.00 [ref] C/T 1.03 [0.87–1.22] T/T 0.98 [0.79–1.23]	0.47	1534/1641	0.36	0.95 [0.85–1.06]	C/C 1.00 [ref] C/T 0.98 [0.82–1.17] T/T 0.90 [0.73–1.12]
+2475 C>A rs2303070	0.07	317 (150/186)	0.05	0.81 [0.65–1.00]	C/C 1.00 [ref] C/A 0.85 [0.68–1.06] A/A 0.15 [0.02–1.13]	0.08	1479/1513	0.06 2 df	0.91 [0.73–1.12]	C/C 1.00 [ref] C/A 0.82 [0.66–1.04] A/A 2.51 [0.81–7.79]
+2915 A>G ss28514856	0.04	184 (92/105)	0.35	0.88 [0.66–1.16]	A/A 1.00 [ref] A/G 0.87 [0.65–1.17] G/G 0.82 [0.22–3.03]	0.03	1547/1685	0.91	0.98 [0.72–1.33]	A/A 1.00 [ref] A/G 0.98 [0.72–1.33] G/G -

MAF: Minor allele frequency, T: transmitted, NT: not transmitted, TDT: transmission/disequilibrium test *P *value, RR: relative risk, 95% CI: 95% confidence intervals, OR: odds ratio N: number.

**Table 3:** Association analyses in type 1 diabetes families and case-control sample sets for rheumatoid arthritis, inflammatory bowl disease and multiple sclerosis associated polymorphisms.

Published SNP	Set 1 and 2 families					Cases and controls				
	MAF	N parent-child trios (T/NT)	P_TDT_	Allelic RR [95% CI]	Genotype RR [95% CI]	MAF	N Cases/controls	P_1df_	Allelic OR [95%CI]	Genotype OR [95%CI]

*IL1RN *+2017 T>C rs2419598	0.28	1418 (916/865)	0.23	1.06 [0.96–1.16]	T/T 1.00 [ref] T/C 1.12 [0.99–1.25] C/C 1.03 [0.83–1.28]	n/a	n/a	n/a	n/a	n/a
*IL1RA *-889 C>T rs1800587	0.30	1431 (925/913)	0.78	1.01 [0.92–1.11]	C/C 1.00 [ref] C/T 1.02 [0.91–1.15] T/T 1.02 [0.83–1.25]	n/a	n/a	n/a	n/a	n/a
*CARD15*										
SNP8 +2023C>T rs2066844	0.03	252 (123/143)	0.22	0.86 [0.68–1.09]	C/C 1.00 [ref] C/T 0.90 [0.70–1.15] T/T 0.22 [0.03–1.66]	n/a	n/a	n/a	n/a	n/a
SNP12 +3641G>C rs2066845	0.01	127 (57/72)	0.19	0.79 [0.56–1.12]	G/G 1.00 [ref] G/C 0.81 [0.57–1.14] C/C -	n/a	n/a	n/a	n/a	n/a
SNP13 +2936insC ss28514842	0.01	143 (73/72)	0.93	1.01 [0.73–1.40]	1/1 1.00 [ref] 1/2 1.00 [0.72–1.38] 2/2 2.99 [0.19–48.22]	n/a	n/a	n/a	n/a	n/a
*IGR2198 *G>C rs11739135	0.40	1511 (988/1008)	0.65	0.98 [0.90–1.07]	G/G 1.00 [ref] G/C 0.95 [0.84–1.07] C/C 0.98 [0.82–1.17]	n/a	n/a	n/a	n/a	n/a
*SLC22A4 *G>A rs3792876	n/a	n/a	n/a	n/a	n/a	0.08	3303/3558	0.93	0.99 [0.87–1.14]	C/C 1.0 [ref] C/T 1.01 [0.87–1.16] T/T 0.83 [0.40–1.70]
*SLC22A4 *C>T rs2073838	n/a	n/a	n/a	n/a	n/a	0.08	3290/3549	0.79	0.98 [0.86–1.12]	G/G 1.00 [ref] G/A 1.00 [0.87–1.15] A/A 0.74 [0.36–1.52]
*LAG3 *G>A rs870849	n/a	n/a	n/a	n/a	n/a	0.36	3860/4297	0.90	1.00 [0.93–1.06]	G/G 1.00 [ref] G/A 1.00 [0.91–1.10] A/A 0.99 [0.86–1.14]

MAF: Minor allele frequency, T: transmitted, NT: not transmitted, TDT: transmission/disequilibrium test *P *value, RR: relative risk, 95% CI: 95% confidence intervals, OR: odds ratio, N: number, n/a: not attempted

As a potential atopy/asthma susceptibility locus, *ADAM33 *was considered as a candidate gene for T1D because atopic illness and T1D have been inversely associated [34]. No functional candidate SNPs in *ADAM33 *have been identified, but it is plausible that the SNPs in introns or the 3' region are located in regulatory sequences and thus may affect transcriptional efficiency or transcript stability [35]. We genotyped six SNPs that showed a *P *≤ 0.03 in asthma case-control data [35] in set 1 families (Tables 1 and 4). One SNP, *ADAM33 *V4 (rs2787094), showed *P *= 0.0004 (RR = 0.78, 95% CI = 0.67–0.89) initially in 754 families, and *P = *4.4 × 10^-6 ^(RR = 0.77, 95% CI = 0.69–0.86) when genotyped in the additional set 2 families. However, we did not obtain additional support for association in 4,326 cases and 4,610 controls (*P = *0.57) (Table 4).

**Table 4:** Association analyses in type 1 diabetes families and case-control sample sets for *ADAM33 *SNPs

Published SNP	Set 1 families					Cases and controls				
	MAF	N parent-child trios (T/NT)	P_TDT_	Allelic RR [95% CI]	Genotype RR [95% CI]	MAF	N cases/controls	P_1df_	Allelic OR [95%CI]	Genotype OR [95%CI]

ST+4 rs44707	0.39	703 (421/443)	0.02*	0.95 [0.83–1.09]	C/C 1.00 [ref] A/C 0.80 [0.67–0.96] A/A 0.99 [0.76–1.30]	n/a	n/a	n/a	n/a	n/a
**V4 rs2787094	0.23	653 (350/451)	0.0004	0.78 [0.67–0.89]	G/G 1.00 [ref] G/C 0.78 [0.67–0.92] C/C 0.59 [0.41–0.85]	0.22	4326/4610	0.57	1.02 [0.95–1.10]	G/G 1.00 [ref] G/C 1.04 [0.95–1.14] C/C 0.99 [0.81–1.22]
Q-1 rs612709	0.14	402 (219/247)	0.02*	0.89 [0.74–1.06]	G/G 1.00 [ref] G/A 0.99 [0.81–1.21] A/A 0.42 [0.22–0.83]	n/a	n/a	n/a	n/a	n/a
ST+7 rs574174	0.20	669 (392/393)	0.97	1.00 [0.87–1.15]	T/T 1.00 [ref] T/C 1.02 [0.87–1.20] C/C 0.92 [0.63–1.34]	n/a	n/a	n/a	n/a	n/a
T+1 rs2280089	0.13	512 (274/304)	0.21	0.90 [0.77–1.06]	G/G 1.00 [ref] C/G 0.90 [0.75–1.07] C/C 0.83 [0.49–1.43]	n/a	n/a	n/a	n/a	n/a
T2 rs2280090	0.13	520 (277/314)	0.13	0.88 [0.75–1.04]	T/T 1.00 [ref] T/C 0.88 [0.73–1.05] C/C 0.81 [0.47–1.39]	n/a	n/a	n/a	n/a	n/a

MAF: minor allele frequency. T: transmitted. NT: not transmitted. TDT: transmission/disequilibrium test. *P *value. RR: relative risk. 95% CI: 95% confidence intervals. OR: odd ratios. N: number. n/a: not attempted.

* Two degree of freedom GTRR (genotype relative risk) *P *value is reported as its significantly different to the TDT *P *value.

** In set 1 and 2 families (1075(571/737) parent-child trios) for *ADAM33 *V4, *P*_TDT _= 4.43 × 10^-6^, with allelic RR = 0.77 [0.69–0.86] and genotype relative risks: G/G 1.00 [ref], G/C 0.80 [0.70–0.91], C/C 0.54 [0.40–0.73].

Finally, we genotyped the *NFKB1 *dinucleotide repeat (CA) microsatellite polymorphism, which has been associated with T1D [36], albeit inconsistently [37] (Table 1). In our set 1 families, we failed to find any evidence for an association with T1D (*P *= 0.68).

## Discussion

Co-localization and overlapping of genetic loci in autoimmune diseases suggests that in some cases, common biological pathways may be involved in the aetiology of T1D and other clinically distinct immune-mediated diseases. In this study we examined 16 genes implicated in autoimmune and other immune-mediated diseases and report that none of the variants tested, consistently showed *P *values of less than 0.05 in association tests with T1D. Our data indicate that, although common immune-mediated disease loci are present in the genome, there are disease genes that are distinct to certain diseases. Indeed, the known T1D susceptibility loci follow this observation: while both *CTLA4 *and *PTPN22 *loci are associated with several autoimmune diseases [3,38], the insulin VNTR locus is likely to be T1D-specific rather than a general autoimmune locus [7].

## Conclusion

The possibility remains that some of the investigated genes and variants are associated with T1D, albeit with weak genetic effects, such as with ORs of less than 1.3, for which the sample size employed in our study does not provide sufficient statistical power. Even a case-control sample of 8,000 cases and 8,000 controls would only have 48% statistical power at a type 1 error rate α of 0.001 for a disease variant with a MAF of 0.10 and OR of 1.15. As genotyping costs decrease, it will be necessary to test the variants in larger sample sets than reported here, because the identification of genetic effects with ORs in the 1.15–1.25 range can be instrumental in the understanding of the disease process of T1D [3]. For example, we note that an observed genetic effect of OR 1.15, such as that exerted by the T1D susceptibility locus *CTLA4*, does not reflect the importance of the biological effect contributed by the locus through the protein(s) it encodes and the pathways it regulates [3].

Lack of association with variants tested here may also be partly due to false positive results obtained in the reported primary disease association studies. As shown in Table 1, these generally employed small sample sets, which is a common explanation in the reporting of *P *values that cannot be replicated in independent studies using larger sample sizes [39-42]. Nevertheless, for ORs >1.5 our present study had good statistical power even for alleles at 0.01 frequency.

## Abbreviations

T1D-type 1 diabetes, SNPs-single nucleotide polymorphisms, MAF-minor allele frequency, OR-odds ratio, RR-relative risk, RA-rheumatoid arthritis, SLE-systemic lupus erythemastosus, GD-Graves' disease, PA-psoriatic arthritis, MS-multiple sclerosis.

## Competing interests

The author(s) declare that they have no competing interests.

## Authors' contributions

DJS performed sequencing, SNP genotyping, data analysis and drafted the manuscript. JMMH performed statistical analysis and drafted the manuscript. FP performed sequencing, SNP genotyping and data analysis. LMM performed sequencing, SNP genotyping, data analysis and drafted the manuscript. JDC performed statistical analysis. KH & CL performed sequencing and SNP genotyping. JH, RB, AV & ID performed SNP genotyping and data analysis.

ACL coordinated annotation of genes. SN prepared DNA samples. NMW managed the data. RCJT coordinated the study. JAT participated in the conception, design and coordination of the study and drafted the manuscript. All authors read and approved the final manuscript.

## Pre-publication history

The pre-publication history for this paper can be accessed here:



## Supplementary Material

Additional File 1 Power calculations for a range of allele frequencies. Click here for file

Additional File 2 Polymorphisms identified in *CRP*. Click here for file

Additional File 3 Polymorphisms identified in *FCER1B*. Click here for file
