# Supplementary material for: Analysis of polymorphisms in 16 genes in type 1 diabetes that have been associated with other immune-mediated diseases
Source: BMC Med Genet. 2006 Mar 6;7:20. doi: 10.1186/1471-2350-7-20 (PMC1420277; doi:10.1186/1471-2350-7-20)
Supplement: Additional File 1 — Power calculations for a range of allele frequencies. [file 1471-2350-7-20-S1.doc]

Additional file 1: Power calculations. Allele frequencies ranged from 48% through to 1% in our dataset. All assumes a multiplicative mode of inheritance.

| 900 trios | | | | | | 1500 cases and 1500 controls | | | | | |
| --- | --- | --- | --- | --- | --- | --- | --- | --- | --- | --- | --- |
| RR | MAF (%) | α | Power (%) | α | Power (%) | OR | MAF (%) | α | Power (%) | α | Power (%) |
| 1.3 | 45 | 0.01 | 91.3 | 0.05 | 97.6 | 1.3 | 45 | 0.01 | 99.4 | 0.05 | 99.9 |
| 1.3 | 40 | 0.01 | 91.2 | 0.05 | 97.6 | 1.3 | 40 | 0.01 | 99.3 | 0.05 | 99.9 |
| 1.3 | 38 | 0.01 | 91.0 | 0.05 | 97.5 | 1.3 | 38 | 0.01 | 99.2 | 0.05 | 99.9 |
| 1.3 | 30 | 0.01 | 88.5 | 0.05 | 96.6 | 1.3 | 30 | 0.01 | 98.6 | 0.05 | 99.7 |
| 1.3 | 28 | 0.01 | 87.4 | 0.05 | 96.1 | 1.3 | 28 | 0.01 | 98.2 | 0.05 | 99.7 |
| 1.3 | 20 | 0.01 | 79.3 | 0.05 | 92.4 | 1.3 | 20 | 0.01 | 95.0 | 0.05 | 98.8 |
| 1.3 | 13 | 0.01 | 63.0 | 0.05 | 83.0 | 1.3 | 13 | 0.01 | 84.3 | 0.05 | 94.8 |
| 1.3 | 10 | 0.01 | 51.6 | 0.05 | 74.6 | 1.3 | 10 | 0.01 | 73.7 | 0.05 | 89.4 |
| 1.3 | 7 | 0.01 | 37.0 | 0.05 | 61.3 | 1.3 | 7 | 0.01 | 56.7 | 0.05 | 78.4 |
| 1.3 | 4 | 0.01 | 20.0 | 0.05 | 41.3 | 1.3 | 4 | 0.01 | 32.5 | 0.05 | 56.4 |
| 1.3 | 1 | 0.01 | 4.5 | 0.05 | 14.3 | 1.3 | 1 | 0.01 | 6.7 | 0.05 | 19.2 |
|  |  |  |  |  |  |  |  |  |  |  |  |
| 1.5 | 45 | 0.01 | 100 | 0.05 | 100 | 1.5 | 45 | 0.01 | 100 | 0.05 | 100 |
| 1.5 | 40 | 0.01 | 100 | 0.05 | 100 | 1.5 | 40 | 0.01 | 100 | 0.05 | 100 |
| 1.5 | 38 | 0.01 | 100 | 0.05 | 100 | 1.5 | 38 | 0.01 | 100 | 0.05 | 100 |
| 1.5 | 30 | 0.01 | 100 | 0.05 | 100 | 1.5 | 30 | 0.01 | 100 | 0.05 | 100 |
| 1.5 | 28 | 0.01 | 100 | 0.05 | 100.0 | 1.5 | 28 | 0.01 | 100 | 0.05 | 100 |
| 1.5 | 20 | 0.01 | 99.8 | 0.05 | 100.0 | 1.5 | 20 | 0.01 | 100 | 0.05 | 100 |
| 1.5 | 13 | 0.01 | 98.5 | 0.05 | 99.7 | 1.5 | 13 | 0.01 | 99.9 | 0.05 | 100 |
| 1.5 | 10 | 0.01 | 95.7 | 0.05 | 99.0 | 1.5 | 10 | 0.01 | 99.4 | 0.05 | 99.9 |
| 1.5 | 7 | 0.01 | 87.0 | 0.05 | 96.0 | 1.5 | 7 | 0.01 | 96.3 | 0.05 | 99.2 |
| 1.5 | 5 | 0.01 | 73.1 | 0.05 | 89.3 | 1.5 | 5 | 0.01 | 88.0 | 0.05 | 96.3 |
| 1.5 | 4 | 0.01 | 61.9 | 0.05 | 82.4 | 1.5 | 4 | 0.01 | 79.1 | 0.05 | 92.2 |
| 1.5 | 1 | 0.01 | 13.4 | 0.05 | 31.5 | 1.5 | 1 | 0.01 | 20.6 | 0.05 | 41.5 |
|  |  |  |  |  |  |  |  |  |  |  |  |
| 2.0 | 45 | 0.01 | 100 | 0.05 | 100 | 2.0 | 45 | 0.01 | 100 | 0.05 | 100 |
| 2.0 | 40 | 0.01 | 100 | 0.05 | 100 | 2.0 | 40 | 0.01 | 100 | 0.05 | 100 |
| 2.0 | 38 | 0.01 | 100 | 0.05 | 100 | 2.0 | 38 | 0.01 | 100 | 0.05 | 100 |
| 2.0 | 30 | 0.01 | 100 | 0.05 | 100 | 2.0 | 30 | 0.01 | 100 | 0.05 | 100 |
| 2.0 | 28 | 0.01 | 100 | 0.05 | 100 | 2.0 | 28 | 0.01 | 100 | 0.05 | 100 |
| 2.0 | 20 | 0.01 | 100 | 0.05 | 100 | 2.0 | 20 | 0.01 | 100 | 0.05 | 100 |
| 2.0 | 13 | 0.01 | 100 | 0.05 | 100 | 2.0 | 13 | 0.01 | 100 | 0.05 | 100 |
| 2.0 | 10 | 0.01 | 100 | 0.05 | 100 | 2.0 | 10 | 0.01 | 100 | 0.05 | 100 |
| 2.0 | 7 | 0.01 | 100 | 0.05 | 100 | 2.0 | 7 | 0.01 | 100 | 0.05 | 100 |
| 2.0 | 4 | 0.01 | 99.9 | 0.05 | 100 | 2.0 | 4 | 0.01 | 100 | 0.05 | 100 |
| 2.0 | 1 | 0.01 | 65.1 | 0.05 | 84.9 | 2.0 | 1 | 0.01 | 73.7 | 0.05 | 88.9 |

MAF: Minor allele frequency,RR: relative risk, OR: odd ratios, α: type 1 error rate
