# Supplementary material for: Analysis of polymorphisms in 16 genes in type 1 diabetes that have been associated with other immune-mediated diseases
Source: BMC Med Genet. 2006 Mar 6;7:20. doi: 10.1186/1471-2350-7-20 (PMC1420277; doi:10.1186/1471-2350-7-20)
Supplement: Additional File 2 — Polymorphisms identified in CRP. [file 1471-2350-7-20-S2.doc]

Additional File 2:

Map positions, location, allele frequency and allelic

R^2^ values for SNPs from *CRP*

| ***Variant ID/ dbSNP*** | ***Map Position*** **On 1q21-23** | ***Location*** | ***MAF*** | ***Allelic R^2^*** |
| --- | --- | --- | --- | --- |
| rs3093077 | 156492709 | 3’ | 0.06 | 99.26 |
| rs3093075 | 156492986 | 3’ | 0.06 | 99.26 |
| rs3093070 | 156493890 | 3’ | 0.02 | - |
| rs2808630 | 156493941 | 3’ | 0.30 | tag |
| rs3093068 | 156494437 | 3’ | 0.06 | 99.26 |
| rs1205 | 156495306 | 3’ | 0.30 | tag |
| ss28514826* | 156495358 | 3’ | 0.02 | - |
| ss28514827* | 156495705 | 3’ UTR | 0.02 | - |
| ss28514828* | 156495743 | 3’ UTR | 0.02 | - |
| rs1130864 | 156496164 | 3’ UTR | 0.30 | 90.38 |
| rs1800947 | 156496511 | exon 2 | 0.08 | tag |
| ss28514831 | 156497122 | intron | Microsatellite | - |
| rs1417938 | 156497259 | intron | 0.25 | tag |
| rs3091244 | 156497738 | 5’ | 0.29 | tag |
| ss28514834* | 156498068 | 5’ | 0.02 | - |
| rs2794521 | 156498169 | 5’ | 0.30 | 95.23 |
| rs3093059 | 156498209 | 5’ | 0.06 | 99.26 |
| ss28514837* | 156500059 | 5’ | 0.30 | tag |
| rs2369251 | 156500319 | 5’ | 0.27 | 86.3 |
| rs6427490 | 156500446 | 5’ | 0.39 | tag |

MAF- minor allele frequency from 32 type 1 diabetes individuals, map position based on NCBI build 35, tag- tag SNP used for genotyping, * indicates novel SNP, Allelic R^2^  value- this is > 80.00 for SNPs that will be captured by the tag SNPs
