# Supplementary material for: Analysis of polymorphisms in 16 genes in type 1 diabetes that have been associated with other immune-mediated diseases
Source: BMC Med Genet. 2006 Mar 6;7:20. doi: 10.1186/1471-2350-7-20 (PMC1420277; doi:10.1186/1471-2350-7-20)
Supplement: Additional File 3 — Polymorphisms identified in FCER1B. [file 1471-2350-7-20-S3.doc]

Additional File 3:

Map positions, location, allele frequency and allelic

R^2^ values for SNPs from *FCER1B*.

|  |  |  |  |  |
| --- | --- | --- | --- | --- |
| ***dbSNP number*** | **Map position**  **on 11q13** | ***Location*** | ***MAF*** | ***Allelic R^2^*** |
| ss28514786* | 5961007 | 5UTR | 0.005 | - |
| ss28514787* | 59610402 | 5UTR | 0.005 | - |
| rs2847659 | 59610428 | 5UTR | 0.42 | 81.095 |
| rs2583477 | 59610431 | 5UTR | 0.47 | tag |
| ss28514790* | 59611667 | 5UTR | 0.01 | - |
| ss28514791* | 59611822 | 5UTR | 0.01 | - |
| rs573790 | 59611961 | 5UTR | 0.34 | tag |
| rs574700 | 59612059 | 5UTR | 0.02 | - |
| rs1441585 | 59612287 | 5UTR | 0.02 | - |
| rs1441586 | 59612604 | 5UTR | 0.44 | 93.153 |
| ss28514796* | 59612898 | intron 1 | 0.01 | - |
| rs2847662 | 59613257 | intron 1 | 0.02 | - |
| ss28514798* | 59613397 | intron 1 | 0.005 | - |
| ss28514799* | 59613956 | intron 2 | 0.005 | - |
| ss28514800* | 59614031 | intron 2 | 0.005 | - |
| rs2583476 | 59614157 | intron 2 | 0.41 | tag |
| rs2847663 | 59614612 | intron 3 | 0.43 | 89.842 |
| ss28514803* | 59615620 | intron 3 | 0.005 | - |
| rs502581 | 59616754 | intron 3 | 0.46 | 95.223 |
| ss28514805* | 59616934 | intron 4 | 0.01 | - |
| ss28514806* | 59617154 | intron 4 | 0.01 | - |
| ss28514807 | 59617853 | intron 4 | Microsatellite | - |
| rs2583471 | 59618390 | intron 6 | 0.41 | 99.003 |
| ss28514808* | 59619606 | exon 7 | 0.005 | - |
| ss28514809* | 59619648 | exon 7 | 0.01 | - |
| rs11230141 | 59621723 | 3UTR | 0.06 | 90.913 |
| rs2847655 | 59622247 | 3UTR | 0.42 | 92.215 |
| ss28514812* | 59622409 | 3UTR | 0.02 | - |
| rs502419 | 59622751 | 3UTR | 0.44 | 95.207 |
| rs2855017 | 59622885 | 3UTR | 0.44 | 94.186 |
| ss28514815* | 59623955 | 3UTR | 0.43 | 95.228 |
| rs12576889 | 59624157 | 3UTR | 0.05 | tag |
| ss28514817* | 59624482 | 3UTR | 0.01 | - |
| rs574704 | 59624489 | 3UTR | 0.5 | tag |
| ss28514819* | 59625068 | 3UTR | 0.006 | - |

MAF- minor allele frequency from 96 type 1 diabetes individuals, map position based on NCBI build 35, tag- tag SNP used for genotyping, * indicates novel SNP, Allelic R^2^  value- this is > 80.00 for SNPs that will be captured by the tag SNPs.
